# Ethnopharmacology Study of Plants from Atlantic Forest with Leishmanicidal Activity

**DOI:** 10.1155/2019/8780914

**Published:** 2019-02-05

**Authors:** Beatriz Mendes Santos, Adriana Bezerra-Souza, Sonia Aragaki, Eliana Rodrigues, Eric Umehara, João Henrique Ghilardi Lago, Márcia Dalastra Laurenti, Susan Pereira Ribeiro, Luiz Felipe Domingues Passero

**Affiliations:** ^1^São Paulo State University (UNESP), Institute of Biosciences, São Vicente, Praça Infante Dom Henrique, s/n, 11330-900 São Vicente, SP, Brazil; ^2^São Paulo State University (UNESP), Institute for Advanced Studies of Ocean, São Vicente. Av. João Francisco Bensdorp, 1178, 11350-011 São Vicente, SP, Brazil; ^3^Instituto de Botânica do Estado de São Paulo, Núcleo de Pesquisa Curadoria do Herbário, Av. Miguel Stefano 3687, CEP 04301-902, São Paulo, SP, Brazil; ^4^Center for Ethnobotanical and Ethnopharmacological Studies (CEE), Institute of Environmental Sciences, Chemical and Pharmaceutical, Universidade Federal de São Paulo (UNIFESP), Brazil; ^5^Centro de Ciências Naturais e Humanas, Universidade Federal do ABC, Avenida dos Estados, 5001 - 09210-580 Santo Andre, SP, Brazil; ^6^Laboratory of Pathology of Infectious Diseases, Medical School, University of São Paulo, São Paulo, SP, Av. Dr. Arnaldo, 455, Cerqueira César, SP, 01246-903, Brazil; ^7^Pathology Department, Case Western Reserve University, 2103 Cornell Rd, room 5503, Cleveland, OH 44106, USA

## Abstract

Leishmaniasis is an infectious disease caused by a protozoan belonging to* Leishmania* genus. Different clinical outcomes can be observed depending on the parasite species and patient's health condition. The outcomes can range from single cutaneous lesions to lethal visceral form. The treatment of all forms of leishmaniasis is based on pentavalent antimonials, and, in some cases, the second-line drug, amphotericin B, is used. Beside the toxicity of both classes of drugs, in some areas of the world, parasites are resistant to antimonial. These detrimental features make fundamental the discovery and characterization of new drugs or plant extracts with leishmanicidal effects. Brazil is a well-known country for its biodiversity. Additionally, the common knowledge inherited for generations in small villages makes Brazil a source of new information and resources for the discovery and development of new drugs. Based on ethnopharmacology, elderlies were interviewed about plants they commonly used for skin diseases and infections. Five native plants from Atlantic forest were indicated; EtOH and* n*-hexane extracts were prepared with the vegetative organs of the plants and assayed against promastigote and amastigote forms of* L. (L.) amazonensis*. The major molecules of each extract were detected using qualitative nuclear magnetic resonance. Among all tested extracts, the* n*-hexane extract from the leave of* Eugenia uniflora *(Myrtaceae), enriched in myricitrin and quercitrin flavonoids, was the most effective against* L. (L.) amazonensis *amastigotes. This data supports the ethnopharmacology approach as a successful tool for the discovery of new drugs with leishmanicidal effects.

## 1. Introduction

Leishmaniasis is endemic in 98 countries accounting for 1.2 million new human infections per year. It is estimated that 90% of the cases are concentrated in poor and marginalized populations of six countries, such as India, Bangladesh, Sudan, South Sudan, Brazil, and Ethiopia [1]. Leishmaniasis is an infectious disease caused by protozoans belonging to the Trypanosomatidae family, Kinetoplastida order and* Leishmania* genus. These parasites infect macrophages from vertebrate hosts, including wild and domestic mammals and humans. The natural vectors of the parasite are invertebrates, belonging to the Diptera order, Psychodidae family,* Lutzomyia* genus in the New World, and* Phlebotomus* genus in the Old World [2]. In the vectors, the parasites survive in the digestive epithelium.

Fourteen* Leishmania* species infect vertebrate hosts. The different clinical outcomes (cutaneous or visceral leishmaniasis) depend on the parasite species, the skin inoculum size, and the host background [3]. The cutaneous leishmaniasis can be caused by different* Leishmania* species [4–6] leading to a wide spectrum of clinical outcomes ranging from a single localized skin lesion, which can spontaneously heal, to multiple ulcerated or nonulcerated lesions, affecting skin and/or mucosa, requiring a more complex treatment [7]. Visceral leishmaniasis can be caused by* L. infantum *and/or* L. donovani *and is fatal if not properly treated [8].

In the New World, one of the most important species causing the cutaneous form of the disease is* Leishmania (Leishmania) amazonensis*. This parasite can cause single ulcerated lesions and also anergic diffuse cutaneous leishmaniasis (ADCL). Parasite dissemination occurs as a consequence of an inefficient T cell response. Additionally, patients infected with this strain have a poor response to the drugs [9].* L. (Viannia) braziliensis *is known to cause single cutaneous lesions and mucosal leishmaniasis (ML) leading to destructive lesions in cartilage, ear, nose, and palate mucosal tissues being a major public health problem [10].

Despite the wide number of* Leishmania* species affecting humans and its different clinical outcomes, treatment is limited, relying just in two compounds, antimonial and amphotericin B. A tartar emetic trivalent antimonial treatment was reported by Gaspar Vianna as highly effective against mucocutaneous leishmaniasis [11]; however, it was retracted as an alternative therapy due to the high toxicity. Pentavalent antimonial therapy remains the drug option as the first-line against all clinical form of leishmaniasis. Nevertheless, the daily parental administration for at least 3 weeks can be detrimental, and the inappropriate drug intake can lead to the emergence of parasite resistance [12, 13]. Additionally, local and systemic side effects, ranging from local pain to acute renal failure and heart toxicity, have been reported.

Amphotericin B, the second-line of leishmaniasis drug, is a polyene antibiotic isolated from the bacteria* Streptomyces nodosus*. It is used as an alternative therapy for antimony treatment resistance/failure [14, 15]. In mammalian cells, amphotericin B interacts with the bilipidic membrane layers forming pores, leading to the loss of osmotic regulation [16, 17]. This causes adverse events including fever, rigors, hypertension/hypotension, hypoxia, and renal and gastrointestinal toxicities [18]. These side effects can be overcome by the liposomal formulation (Ambisome). Despite the low toxicity of this formulation, the high costs limit its use in low-income countries, the majority of the endemic regions. Based on the limitations of the current available drugs against leishmaniasis, the search for more effective, less toxic, and inexpensive drugs is vital. In the present work we took advantage of the Brazilian biodiversity [19] and its rich native common knowledge about plants used for medicinal purposes to generate extracts and test it against* Leishmania* parasites.

The Brazilian Atlantic forest covers around 65,000 km^2^ from 14 states of the country. It is the richest forest in the world in wood plants per unit area. Interestingly different ethnic groups live in this region, such as South American natives, “quilombolas” (descendants of Afro-Brazilian people), miscegenation of Indian, African, European, and Asian people. These groups have an extensive common knowledge on medicinal plants and its usage. Ethnopharmacological survey questionnaire on plants and animals utilized by migrants living in Atlantic forest in São Paulo state (Brazil) with medicinal purposes was developed by Garcia et al. [20]. The use of this questionnaire leads to the report of 12 animals and 85 plants used for different purposes.

Based on the stated above, the major goal of the present study was to analyze the leishmanicidal effect of plant extracts indicated by elderly people living in the coastal zone of São Paulo state.

## 2. Material and Methods

### 2.1. Data Collection

This study was approved by the Ethics Committee of São Paulo State University (Plataforma Brasil N^o^ 68501817.6.0000.5398) and by Ministério do Meio Ambiente (SISBio No. 57877). The interviewees also signed consent forms granting permission to access their knowledge.

Data collection was performed by São Paulo State University, São Vicente, SP, Brazil, between April and June 2017. Sixteen elderly women from “*Universidade Aberta da Terceira Idade* – UNATI” (Open University of the Elderly) were interviewed. São Vicente is in the bay area of Santos with estimated 363.173 habitants, covering an area of 148,100 km^2^, and the Atlantic forest biome covers part of this city.

The interview consisted of gathering knowledge about the usage of medicinal plants for different purposes, especially the ones used for treating skin problems and infections. Only native plants of Atlantic forest were selected to be investigated in the present study.

Personal and ethnopharmacological data from the interviewees were obtained by the authors (B. Mendes and L.F.D. Passero) through informal and semistructured interviews [20] that addressed the following topics: personal details (name, sex, age, ethnicity, main occupation, grade of schooling, source of knowledge, health condition, and income) as well as ethnopharmacological data (vernacular name of the plant, its use, part used, formula, route of administration, contraindications, dosages, and restrictions).

The indicated medicinal plants were collected in the presence of the person who described it during the interviews, in accordance with the methods suggested by Fidalgo & Bononi [21]. The scientific names were determined by specialists from Instituto de Botânica do Estado de São Paulo (IBt), and vouchers were deposited at its Herbaria.

Forty-six plants to treat different health problems were indicated; however, just five species were selected for further characterization based on their application for skin problems or infections. The scientific identifications are* Alternanthera brasiliana *(L.) Kuntze (Amaranthaceae) (voucher number LFP01),* Eugenia uniflora* L. (Myrtaceae) (voucher number LFP02),* Jatropha gossypiifolia* (Euphorbiaceae) (voucher number LFP03),* Schinus terebinthifolia *Raddi (Anacardiaceae) (voucher number LFP04), and* Stachytarpheta cayennensis *(Verbenaceae) (voucher number LFP05). This last plant was not analyzed in the present study, since its action on* L. (L.) amazonensis *was already analyzed previously [22].

### 2.2. Production of Extracts

Dried and powdered organs of plants, such as leaves or fruits (18 g), were extracted with* n*-hexane (8 X 20 mL) and subsequently with EtOH (10 X 20 mL) at room temperature. The obtained materials were evaporated under reduced pressure to afford dried crude extracts. All extracts were dissolved in DMSO and filtered in 0.22 *μ*m to study the biological activities and the adequate solvent for chemical characterization. All extracts were stored at -20°C.

### 2.3. Parasites

*L. (L.) amazonensis* parasite (MHOM/BR/73/M2269) was kindly provided by Prof. Dr. Fernando T. Silveira from the cryobank of “Leishmaniasis Laboratory Prof. Dr. Ralph Laison”, Department of Parasitology, Evandro Chagas Institute, Ministry of Health, Belém, Pará, Brazil. The parasite was identified using monoclonal antibodies and isoenzyme electrophoretic profiles at the Leishmaniasis Laboratory of the Evandro Chagas Institute (Belém, Pará state, Brazil). Parasites were grown in Schneider medium, supplemented with 10% heat-inactivated fetal bovine serum, 10 *μ*g/mL of gentamicin, 1000 U/mL of penicillin, supplemented with 10% heat-inactivated fetal calf serum (Gibco), 0.25% hemin (Sigma-Aldrich) and 2% sterile male human urine at 25°C (S10 medium). Promastigote forms in the stationary phase were used.

### 2.4. Evaluation of Antipromastigote Effect of Plant Extracts

Promastigote forms of* L. (L.) amazonensis* (2x10^6^ promastigotes/well) were incubated in 96-well culture plate in S10 medium with* n*-hexane and EtOH extracts in a range from 4x10^−1^ to 100 *μ*g/mL. The standard drug miltefosine was added in culture in the range from 4x10^−1^ to 100 *μ*g/mL. Negative control group was cultivated in medium and DMSO as vehicle solution (never exceeding 1% v/v). The parasites were incubated for 24h at 25°C. Then, the parasites were washed with 200 *μ*L of sodium chloride 0.9% (w/v) three times with centrifugation at 3000 rpm, 10 min at 4°C, followed by addition of MTT (3-(4,5-dimethylthiazol-2-yl)-2,5-diphenyltetrazolium bromide) (4 mg/mL). Four hours later, 50 *μ*L of 10% sodium dodecyl sulfate (SDS) was added to each well. The plates were further incubated for 18h and read in ELISA reader at 595 nm. Effective concentration 50% (EC_50_) was estimated using Graph Pad Prism 5.0 software.

### 2.5. Cytotoxicity Studies

The macrophage cell lineage J774 (5 x 105 cell/well) were cultured in RPMI 1640 medium supplemented with 10% heat-inactivated fetal bovine serum, 10 *μ*g/mL of gentamicin, and 1,000 U/mL of penicillin (R10 medium) with extracts or miltefosine (4x10^−1^ to 100 *μ*g/mL). As negative control, macrophages were cultivated in medium and DMSO as vehicle solution (never exceeding 1% v/v). After 24h, cell viability was analyzed by MTT method. Cytotoxic concentration 50% (CC_50_) was estimated with Graph Pad Prism 5.0 software.

The selectivity indexes (SI) were calculated using the ratio: CC_50_ /EC_50_ toward promastigote (SIp) or amastigote (SIa) forms, and this number represents how much active is a given plant extract toward the parasites when compared with the host cell.

### 2.6. Macrophage Infection and Treatments

J774 macrophages (5 x 10^5^ macrophage/mL) were cultivated in round cover slips in 24-well plate with R10 medium, followed by infection with* L*.* (L*.*) amazonensis* promastigotes at a ratio of 20 parasites per 1 macrophage. Plates were incubated at 5% CO_2_ at 35°C. After 24 h of culture,* n*-hexane and EtOH extracts were added in three different concentrations ranging from 5 to 40 *μ*g/mL to evaluate the efficacy of these extracts against intracellular parasites [23–26]. Miltefosine was added as the standard drug. After 24h of treatments, round cover slips were dried at room temperature, fixed in methanol, and stained by Giemsa. The Infection Index (II) was estimated [27] according to the following expression:

(1) $$\begin{array}{c} II=\%\;\;of\;\;infected\;\;macrophages\;\;x\left(\;\frac{internalized\;\;amastigote\;\;forms}{macrophages}\;\right) \end{array}$$

The concentration able to decrease the II to 50% was estimated and used to compare the efficacy of extracts with the standard drug miltefosine.

### 2.7. Qualitative Nuclear Magnetic Resonance (NMR) Analysis of Extracts

Crude extracts were analyzed using NMR, especially ^1^H and ^13^C NMR experiments. NMR spectra were recorded on a Bruker Avance III 500 spectrometer, using 5 mm TXI probe, operated at 500 MHz for ^1^H and 125 MHz for ^13^C nuclei, respectively. Approximately 10 mg of each sample was dissolved in 0.6 mL of CDCl_3_ (*n*-hexane extracts) or DMSO-d_6_ (EtOH extracts).

## 3. Results

### 3.1. Characterization of Studied Population and Plants Indicated

**Figure 1:**
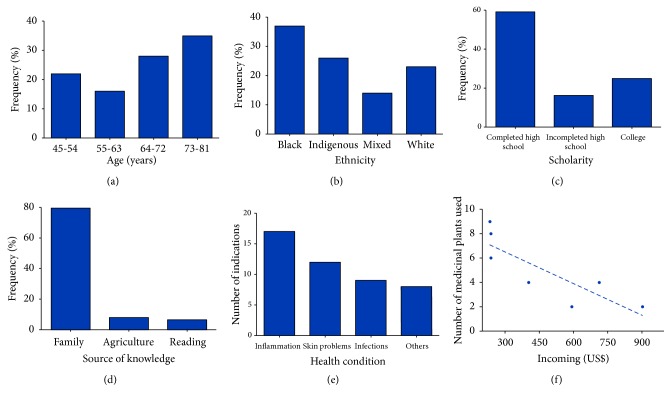
Personal and ethnopharmacological data of interviewees were obtained through informal and semistructured interviews. Age range (a), ethnicity (b), scholarity (c), source of knowledge (d), plants usage (e), and the correlation between income and the number of medicinal plants used by elderlies (f).

The age from the interviewees ranged from 45 to 81 years old. The oldest group (73-81 years old) was the most frequent (35%), followed by 64-72 (28%), 45-54 (21%), and 55-63 years (16%) (Figure 1(a)). Regarding ethnicity, the majority of the participants identified themselves as afro-descendent (37%), followed by indigenous (26%), whites (23%), and mixed (14%) (Figure 1(b)). The majority of them completed high school (59%), 16% have incomplete high school, and 25% of the interviewees have college degree (Figure 1(c)). Eighty-five percent of the participants learned about the medicinal properties of the plants with family, 8.1% learned through working in agriculture, and the remaining by reading (6.6%) (Figure 1(d)). The categories of medicinal plants used were inflammatory process (17 plants), skin problems (12 plants), infections (9 plants), and other health conditions (8 plants) (Figure 1(e)). Due to the easy accessibility to the natural plants versus commercial drugs, an inverse correlation between monthly income and the number of medicinal plants used for pathological conditions was observed. The interviewees with the lowest income used more medicinal plants compared with ones presenting the highest income (Figure 1(f)).

### 3.2. Leishmanicidal and Cytotoxic Assays

Among polar extracts, it was observed that EtOH extract from the leaves of* S. terebinthifolius *was the most active for controlling promastigote forms of* L. (L.) amazonensis *(EC_50_ of 30.5 ± 6.3 *μ*g/mL). The least active compound was the extract from fruits of* S. terebinthifolius *(EC_50_ of 64.6 ± 10.2 *μ*g/mL). EtOH and* n*-hexane extracts from* A. brasiliana *and* J. gossypiifolia *did not show any leishmanicidal effect. In respect to cytotoxic effects, it was observed that EtOH extracts presented CC_50_ above 90 *μ*g/mL (Table 1), and in this case the most selective extract was produced with the leaves of* S. terebinthifolius*.

Nonpolar* n*-hexane extracts from the leaves of* S. terebinthifolius *and* E. uniflora *as well as fruits of* S. terebinthifolius* controlled promastigote forms of* L. (L.) amazonensis *with EC_50_ of approximately 14 *μ*g/mL. N-hexane extracts from the leaves and fruits of* S. terebinthifolius *presented mild cytotoxic. The most selective* n*-hexane extracts were obtained from fruits and leaves of* S. terebinthifolius *(SI of 3.7) and* E. uniflora *(SI of 3.6), respectively. The standard drug, miltefosine, was active against promastigote forms of* L. (L.) amazonensis *with an EC_50_ of 3.2 ± 0.9 *μ*g/mL, mild cytotoxicity (CC_50_ of 38.2 ± 3.3 *μ*g/mL) and a SI of 14.8.

Among polar extracts, the most active against amastigote forms of* L. (L.) amazonensis* was obtained from leaves of* E. uniflora *(CE_50_ = 22.1 ± 5.8 *μ*g/mL); furthermore, the SI of these extract was higher than 4.5. In addition, EtOH extracts from leaves and fruits of* S. terebinthifolia* presented mild activity against intracellular amastigotes (SI > 2). EtOH extracts from leaves of* A. brasiliana *and* J. gossypiifolia *did not show leishmanicidal activity.

Among* n*-hexane extracts, the most active one was obtained from the leaves of* E. uniflora* (CE_50_ = 9.2 ± 1.2 *μ*g/mL) and showed a SI of 5.5, followed by* S. terebinthifolia* extract (CE_50_ = 17.4 ± 1.0 *μ*g/mL; SI = 2.2). The* n*-hexane extract from fruits of* S. terebinthifolia *showed moderated activity toward amastigote forms (Table 1). The extract from leaves of* J. gossypiifolia *did not show antiamastigote activity. These data are summarized in Table 1.

### 3.3. Qualitative Nuclear Magnetic Resonance (NMR) Analysis of Extracts

^1^H NMR spectra of* n*-hexane extracts from leaves and fruits of* S. terebinthifolia* showed characteristic compounds, such as tirucallane type triterpenes. Analysis of ^13^C NMR spectra indicated the presence of (*Z*)-schinol and (*Z*)-masticadienoic acid as the main metabolites. Fruit extract was composed predominantly by unsaturated fatty acids in free form as triacylglycerol derivatives. NMR spectra of both leaves and fruits EtOH extracts indicated the predominance of *β*-glucose.

The* n*-hexane extract from the leaves of* E. uniflora* was essentially composed by unsaturated fatty acids. These spectra showed less intense peaks, which were assigned to the sesquiterpene atractylon. Analysis of the ^1^H NMR spectrum of EtOH extract from* E. uniflora* indicated the predominance of glucosylated flavonoids, especially those containing myricetin and quercetin aglycone moieties. Furthermore, analysis of ^13^C NMR spectrum indicated the predominance of flavonoids myricetin-3-O-*α*-L-rhamnopyranoside (myricitrin) and quercetin-3-O-*α*-L-rhamnopyranoside (quercitrin).

NMR spectral analysis of* n*-hexane extract from leaves of* J. gossypifolia* indicated the predominance of unsaturated fatty acids. The EtOH extract, as indicated by ^13^C NMR spectral analysis, was composed by flavonoids vitexin, isovitexin, orientin, and isoorientin.

## 4. Discussion

Few drugs are available for leishmaniasis treatment and in general present severe side effects and can induce resistance in parasites. This highlights the urgent need for new drugs with leishmanicidal effect: broad spectrum, less toxic, and cost-effective. Natural sources can be an alternative resource for the screening of new compounds with medicinal properties. These new natural sources can be investigated using different strategies, such as random collecting (*at random*), collecting oriented by chemotaxonomy, biorational collecting (guided by chemical ecology), and collecting based on traditional and/or popular knowledge (ethnopharmacology) [28]. The ethnopharmacological is an interesting strategy, since it investigates the biodiversity of flora and/or fauna and the diversity of human behavior in respect to the use of natural resources for different pathological conditions; in fact, traditional people have extensive knowledge on the use of plants and animals as medicaments [29, 30]. Specifically, for leishmaniasis the traditional knowledge in New and Old Worlds has indicated different plant species with leishmanicidal effect [31]. In Brazil, for example, studies reflect this relation, since plants used popularly [32, 33] or by ethnical groups [34] were effective against promastigote and amastigote forms of* L. (L.) amazonensis*,* L. (V.) braziliensis,* and* L. (L.) chagasi*. Few studies have used popular knowledge mainly from elderly subjects to guide the discovery of new natural sources of leishmanicidal activity.

An ethnobotanical study conducted near to Chapada Diamantina National Park [35] showed the importance of elderlies as a fundamental source of knowledge about medicinal plants. The present manuscript corroborated that elderlies have interesting information about plants with medicinal properties. In addition, these group used plants commonly as tea or infusions, and the most cited plants (24 reports) were mint, rosemary, and lemongrass (data not shown). Interestingly, these plants are not natives from Atlantic forest and possibly their use as medicaments can also be related to the miscegenation of Brazilian people and the introduction of plants by immigrants, such as Europeans, Africans, and Asians [36, 37]. In fact, the group studied herein is a mix of different ethnicities (Africans, indigenous, mixed and white people) and thus the use of native and exotic species of plants as alternative medicines is expected.

Most of the knowledge was inherited from past generations [38] and a minor part learned by working in agriculture or reads. Different from traditional communities that prioritize the use of natural resources as medicaments, as observed in several studies conducted by us in Brazil among Quilombolas [39] and Krahô Indians [40], an inverse association between income and number of medicinal plants used was found in the present study. It indicated the limitation in getting commercial available drugs by low-income population, making plant extracts the first choice of treatment [41]. However, another ethnobotanical study conducted in northeast Brazil (“Caatinga” biome) shows high income population, with high knowledge prioritizing the usage of medicinal plants [42]. Thus, in this work the set of ethnographic data collected from elderly people showed they possess a solid knowledge about medicinal plants. Indeed, during the period of interviews they indicated 52 plants (native and not native) for different pathological conditions, which were grouped in four classes of diseases (inflammation, skin problems, infections, and others). Considering the biodiversity of Atlantic forest and the importance of this biome for different ethnic and nonethnic groups we opted to study only native specimens of Atlantic forest and specifically plants indicated to skin diseases or infections.

The extracts of indicated plants presented activity against* L. (L.) amazonensis*, being the* n*-hexane from leaves and fruits of* S. terebinthifolia *and* E. uniflora *the most active ones. Similarly, extracts from these species were also active against amastigote forms, and, in this case,* n*-hexane extract from the leaves of* E. uniflora *was the most active against intracellular amastigote forms, presenting specificity for amastigotes of 5.5 in comparison with host cells. Furthermore, it was also verified the selectivity of EtOH extract from leaves of* E. uniflora*, suggesting this plant species has active molecules and thus should be further investigated. Previous works showed that extracts from plants of genus* Eugenia *were active against promastigote and amastigote forms of* L. (L.) amazonensis* [43, 44]. Additionally, the essential oil from* E. uniflora *affected amastigote forms viability by increasing phagocytic and lysosomal activities of host macrophages [45]. Previous works also related the activity of* S. terebinthifolia *bark extracts on promastigote forms of* L. (L.) amazonensis* [34, 46] as well as purified triterpenes against* L. (L.) infantum *[47].

Extracts from the leaves and fruits of* S. terebinthifolia *were composed by tirucallane type triterpenoids schinol and masticadienoic acid [48] showing activity against intracellular forms of* L. (L.) infantum *as well as against trypomastigote forms of* Trypanosoma cruzi *[47]. In addition, fruits extracts have shown the predominance of *β*-glucose and reduced amounts of (Z)-masticadienoic acid [49], which can account for the lower activity of this extract when compared with the leaves.

*E. uniflora* extracts were composed mainly of the sesquiterpene atractylon [50] glucosylated flavonoids [51]. Among the identified flavonoids, quercitrin has the EC_50_ already reported and its activity on* Leishmania *arginase [52, 53]. No further reports about leishmanicidal activity of the other flavonoids in extracts of* E. uniflora *were observed. These molecules can be considered an interesting target to develop new prototype medicines.

Although not effective in* Leishmania *parasites, the NMR spectra of* J. gossypifolia *extracts were analyzed suggesting the occurrence of flavonoids. Recently, the leishmanicidal activity of the flavonoids isovitexin and vitexin was analyzed against promastigote and amastigote forms of* L. (L.) donovani *and* L. (L.) amazonensis*, being more effective against the visceral than the cutaneous counterpart [54, 55]. Furthermore, orientin-containing fraction from* Cecropia pachystachya* altered* L. (L.) amazonensis* viability by interfering in the mitochondrial-kinetoplast complex and inhibiting parasite arginase enzyme [56]. Although these bioactive compounds were detected in* J. gossypiifolia* extracts [57] in the present manuscript leishmanicidal activity was not observed, what in fact can be related to the concentration of this set of molecules in the extract or even molecular interaction between molecules could inhibit the leishmanicidal activity.

## 5. Conclusions

Data shown in the present manuscript report that extracts produced with* E. uniflora *and* S. terebinthifolia *species have selectivity to promastigote and amastigote forms of* L. (L.) amazonensis*, and this activity can be associated with the major molecules present in the respective crude extracts. In addition, the present manuscript suggests that popular knowledge can be an interesting tool to aid the discovery of new and effective molecules against* Leishmania *sp.

## Figures and Tables

**Table 1:** Leishmanicidal activity of EtOH and *n*-hexane extracts were assayed against promastigote and amastigote forms of *L. (L.) amazonensis*. Cytotoxicity was analyzed using J774 macrophage cell lineage. EC_50_ = effective contraction 50%; CC_50_ = cytotoxic concentration 50%; SIp = selectivity index towards promastigote forms; SIa = selectivity index towards amastigote forms.

**Species (Family)**	**Part indicated**	**Extract**	**EC**_**50**_ ** (**μ**g/mL)** **promastigote**	**CC**_**50**_ ** (**μ**g/mL)** **macrophage**	**EC**_**50**_ ** (**μ**g/mL)** **amastigote**	**SIp**	**SIa**
*A. brasiliana* (Amaranthaceae)	Leaves	EtOH	N/A	≥ 100.0	N/A	-	-
		*n*-hexane	N/A	≥ 100.0	N/A	-	-
*E. uniflora* (Myrtaceae)	Leaves	EtOH	47.0 ± 8.5	≥ 100.0	22.1 ± 5.8	≥ 2.1	≥4.5
		*n*-hexane	14.0 ± 3.3	50.5 ± 7.2	9.2 ± 1.2	3.6	5.5
*J. gossypiifolia* (Euphorbiaceae)	Leaves	EtOH	N/A	≥ 100.0	N/A	-	-
		*n*-hexane	N/A	≥ 100.0	N/A	-	-
*S. terebinthifolia* (Anacardiaceae)	Fruits	EtOH	64.6 ± 10.2	91.4 ± 4.0	30.6 ± 9.6	1.4	2.9
		*n*-hexane	13.9 ± 4.3	52.0 ± 2.4	43.1 ± 10.3	3.7	1.2
*S. terebinthifolia* (Anacardiaceae)	Leaves	EtOH	30.5 ± 6.3	92.2 ± 8.0	32.6 ± 5.1	3.0	2.8
		*n*-hexane	14.7 ± 3.0	38.1 ± 7.3	17.4 ± 1.0	2.6	2.2
Miltefosine	-	-	2.8 ± 0.5	41.3 ± 4.6	6.8 ± 2.1	14.8	6.1

## Data Availability

The data used to support the findings of this study are available from the corresponding author upon request.
